# A Handbook of Diseases of the Eye and Their Treatment

**Published:** 1888-09

**Authors:** 


*A Handbook of Diseases of the Eye and their Treatment.* By HENRY R. SWANZY, A.M., M.B., F.R.C.S.I. Second Edition. London: H. K. LEWIS. 1888.

From frequent reference to the first edition of this book, we are able to say that it contains much more information on the subject upon which it treats than could be expected from its size; and the present improved edition is perhaps, taken all together, the best book of its class extant on diseases of the eye, and it should certainly be in every oculist's library. For the family physician the work contains everything that is likely to be required; but it can scarcely be considered to be fully illustrated, though it contains 150 woodcuts. Symptoms, causes, diagnosis, and prognosis are systematically dealt with under each disease; and the rules for treatment are given fully and definitely, as might be expected from so experienced a clinician as Dr. Swanzy. The chapter on sympathetic ophthalmitis is eminently practical: that on glaucoma deals fully with the symptoms in very simple language, and gives a succinct account of the pathology as at present understood.

The medical side of ophthalmology is well represented from the pathological standpoint, as in the chapter on diseases of the retina, in which it is of such importance to ascertain the condition of the vascular organs, of the kidneys, and of the "diathesis" or disposition of the body: but our author here, as also in writing upon the choroid, gives fewer suggestions for treatment of the eye-lesion than might have been expected from him; although, it must be admitted, more heroic or experimental measures are frequently of no avail. The chapter on the pupil is a short thesis in itself, based upon very wide reading on the subject. The conditions and actions of the orbital muscles, diplopia, squint and its treatment, receive full consideration well up to date.

An appendix gives information as to the standards of vision required for the various Government services.

